# Regional differences of *Mycobacterium tuberculosis* complex infection and multidrug resistance epidemic in Luoyang

**DOI:** 10.1186/s12879-024-09395-w

**Published:** 2024-06-11

**Authors:** Zhenzhen Wang, Tengfei Guo, Liyang Xu, Jinwei Liu, Yi Hou, Junrong Jin, Qing Zhang, Tao Jiang, Zhanqin Zhao, Yun Xue

**Affiliations:** 1 The First Affiliated Hospital and Clinical Medical College, Henan University of Science and Technology, Luoyang, China; 2 Luoyang Center for Disease Control and Prevention, Luoyang, China; 3 College of Animal Science and Technology, Henan University of Science and Technology, Luoyang, China; 4 School of Medical Technology and Engineering, Henan University of Science and Technology, Luoyang, China

**Keywords:** *Mycobacterium tuberculosis* complex, Multidrug resistance, Tuberculosis, Regional differences

## Abstract

**Background:**

Tuberculosis (TB) remains a global public health event of great concern, however epidemic data on TB covering entire areas during the special period of the COVID-19 epidemic have rarely been reported. We compared the dissemination and multidrug-resistance patterns of *Mycobacterium tuberculosis* complex (MTBC) in the main urban area of Luoyang City, China (including six municipal jurisdictions) and nine county and township areas under its jurisdiction, aimed to establish the epidemiology of TB in this region and to provide reference for precision anti-TB in places with similar settings.

**Methods:**

From 2020 to 2022, sputum samples were collected from 18,504 patients with confirmed, suspected and unexcluded TB in 10 designated TB medical institutions. Insertion sequence 6110 was amplified by PCR (*rpoB* gene detection if necessary) to confirm the presence of MTBC. PCR-positive specimens were analyzed by multicolor melting curve analysis to detect multidrug resistance.

**Results:**

Among the 18,504 specimens, 2675 (14.5%) were MTBC positive. The positive rate was higher in the main urban area than in the county and township areas (29.8% vs. 10.9%, *p* < 0.001). Male, re-treated and smear-positive groups were high-burden carriers of MTBC. Individuals aged > 60 years were the largest group infected with MTBC in the main urban area, compared with individuals aged < 61 years in the county and township areas. The detection of multidrug-resistant TB (MDR-TB) was higher in the main urban area than in the county and township areas (13.9% vs. 7.8%, *p* < 0.001). In all areas, MDR-TB groups were dominated by males, patients with a history of TB treatment, and patients aged < 61 years. Stratified analysis of MDR-TB epidemiology showed that MDR4 (INH þ RIF þ EMB þ SM) was predominant in the main urban area, while MDR3 (INH þ RIF þ SM) was predominant in the county and township areas. MDR-TB detection rate and epidemiology differed among the county and township areas.

**Conclusions:**

For local TB control, it is necessary to plan more appropriate and accurate prevention and control strategies according to the regional distribution of MTBC infection.

## Introduction

Tuberculosis (TB) causes millions of deaths every year [1]. Effective prevention and control measures have been established, and it is hoped that the goal of the End TB Strategy will be achieved by 2035 [2]. The road to achieving this goal has many obstacles and unknown factors, such as war, changes in economic situation and population structure, and epidemic outbreaks. This requires continuous update and analysis of the latest and most comprehensive data on *Mycobacterium tuberculosis* complex (MTBC) epidemics and drug resistance, to improve the relevant systems and policies to ensure prevention and control of TB. The incidence of TB in China ranks third among 30 countries with high burden of TB, with about 30,000 multidrug-resistant/rifampin-resistant TB (MDR/RR-TB) patients [1]. High rates of TB drug resistance make us look again at the effectiveness of TB control policies. Real-time knowledge of the unique characteristics of TB incidence and drug resistance can help to better understand the current state of the TB epidemic, which is critical for public health programs and policy makers [3].

Although the burden is still huge, the incidence and mortality of TB have shown a downward trend in the past 10 years, and overall control is moving in the right direction. However, the COVID-19 pandemic has destroyed this positive outlook and has negatively affected confidence in TB prevention and control [4]. During the COVID-19 pandemic, the real number of cases of TB was more than actually reported, which was mainly due to fewer opportunities for visits to TB-designated institutions, delayed diagnosis and treatment, and shortage of medical resources [5]. It is estimated that TB deaths will increase by 20% in the post-COVID-19 period, and co-infection with both diseases is also a risk factor for increased case fatality rates [6–8]. Despite this, the impact of COVID-19 prevention and control measures on TB has not been fully explained, and the drivers of dual mortality between TB and COVID-19 are not fully understood [9]. There are few local reports on the prevalence and drug resistance of TB during the COVID-19 pandemic. As an important industrialized city in China, Luoyang is a heavily affected area for tuberculosis infection and drug resistance [10, 11]. However, more comprehensive information on the spread of tuberculosis in both urban and rural areas has always been scarce. Especially since the first case of COVID-19 appeared in January 2020, there is still a lack of data on the prevalence and drug resistance of MTBC in this special period, which is not conducive to fully assessing the risk to public health or to achieving the goal of the End TB Strategy.

In this study, we analyzed the spread of TB and the prevalence of multidrug-resistant TB (MDR-TB) during the COVID-19 pandemic in the main urban area of Luoyang City, China (including six municipal jurisdictions) and nine county and township areas under its jurisdiction. We summarize the lessons learned for diagnosis and treatment of TB during the COVID-19 pandemic, provide data for formulating TB prevention and control strategies during public health emergencies, and also supplemented the data of public anti-tuberculosis undertakings.

## Methods

### Study area and design

**Fig. 1:**
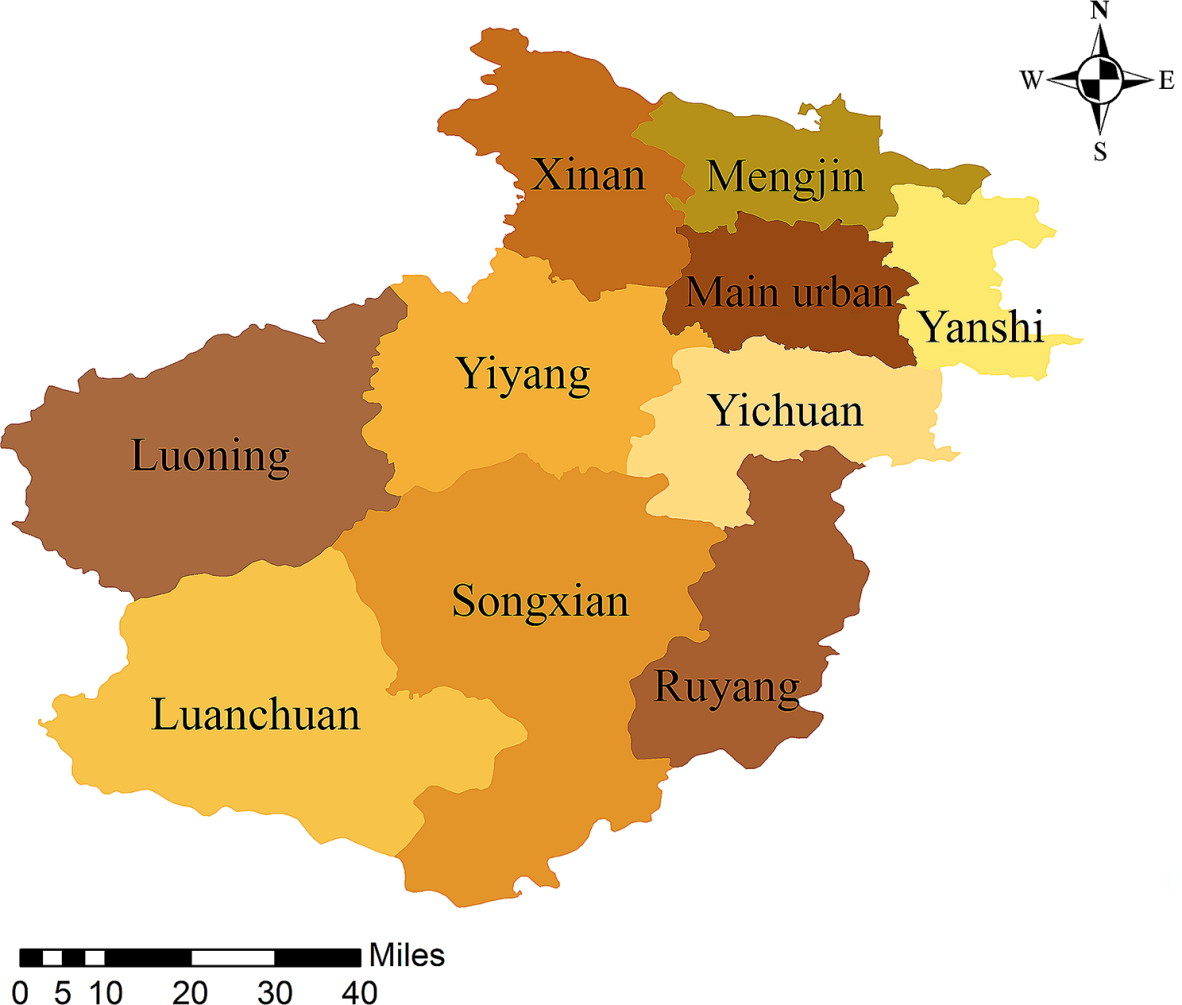
Map of study area. The main urban areas (including six administrative areas) are Laocheng District, Xigong District, Chanhe District, Jianxi District, Jili District and Luolong District, respectively. The nine county and township areas are Mengjin County, Xin’an County, Luanchuan County, Songxian County, Ruyang County, Yiyang County, Luoning County, Yichuan County and Yanshi City, respectively

Henan is the largest province in eastern China, and Luoyang is one of its most important prefecture-level cities, with six municipal districts and nine county and township areas (Fig. 1). In 2021, there was a total area of 15,230 km^2^ and a resident population of 7.056 million, of which the resident population of the main urban area of Luoyang City is 2.48 million, and the county and township areas under its jurisdiction are 4.57 million [12]. The study was conducted from January 2020 to February 2022. Sputum samples were collected from patients attending 10 designated medical and health units for TB in Luoyang City. We evaluated MTBC carriage and MDR-TB in high-risk groups for TB in this area.

### Study population and sample collection

We collected 18,504 sputum samples from the 10 designated TB hospitals. (These were from who actively visited designated TB medical institutions and from cases referred by other institutions after TB screening. According to the specific situation of patients coming to the clinic, TB experts screen eligible cases and issue sputum examination prescriptions to these patients, and the patients submit the samples after collecting the samples as required.). Information such as the sex and regional distribution of patients was obtained from the local TB reporting network and the electronic medical record system at the time of treatment.

### Sample collection and shipment

Sputum samples were obtained according to the Guidelines for the Implementation of the China Tuberculosis Prevention and Control Program issued by the Chinese Center for Disease Control and Prevention [13]. Three consecutive sputum samples (nocturnal, morning and random) were collected from each patient in 50-mL screw cap test tubes, which were sealed and transported to the TB laboratory of Luoyang Infectious Disease Hospital for testing. Transport specimens in strict accordance with the Regulations on the Management of the Transportation of Highly Pathogenic Microorganisms (Viruses) or Samples that Can Infect Humans (Order No. 45 of the former Ministry of Health) [14]. The sample volume was usually 3–5 mL, and the preliminary judgment whether the specimen is qualified according to the traits (caseous sputum, blood sputum and mucous sputum were judged as qualified specimens) .

### Mycobacterial isolation and preliminary strain identification

Sputum samples were stained with acid-fast stain and examined microscopically to determine their quality. Four volumes of 4% NaOH solution were added to all sputum samples and the Sanity 2.0 System (Xiamen Zeesan Biotech Co. Ltd.) was used for DNA extraction and real-time fluorescence PCR analysis. Detection of MTBC-specific insertion sequence 6110 confirmed that the isolate was MTBC, and *rpoB* gene was detected if necessary for strain confirmation. Negative/positive controls were used to control for instrument operation, experimental environment and personnel operation.

### Drug sensitivity and resistance gene analysis

The real-time PCR positive samples were analyzed by multicolor melting curve analysis (MMCA) to detect the molecular loci of first-line anti-TB drug resistance in MDR *M. tuberculosis*. Rifampicin: *rpoB* codon 507–512, *rpoB* codon 521–528, *rpoB* codon 513–520, *rpoB* codon 529–533; isoniazid: *AhpC* promoter region (−44 to −30 and −15 to 3 sites), *inhA* codon 94, *inhA* promoter region (−17 to −8), codon *KatG* 315; ethambutol: *embB* codon 306, *embB* codon 406, *embB* codon 497, *embB* codon 368/378/380; streptomycin: *rpsL* codon 43, *rpsL* codon 88, *rrs* codon 513–517, *rrs* codon 905–908. Nucleic acid templates for molecular susceptibility testing were automatically extracted on Lab-Aid 824 Nucleic Acid Extractor (Xiamen Zeesan). Performed the test in strict accordance with the standard operating procedures required in the package insert of the drug resistance mutation assay kit. Extracted *M. tuberculosis* DNA template (5 µl) was added to 20 µl PCR system for amplification and melting curve analysis in a quantitative fluorescence PCR instrument (shanghai hongshi - SLAN-96P). When the melting temperature (Tm) of the specimen was consistent with that of the positive control (error < 1℃), it was judged as wild type, and when the △Tm value was > 2℃, it was judged as mutant, suggesting that the strain was resistant to this drug. We conducted intra-batch quality control in strict accordance with the operating instructions.

### Quality control

The quality procedures for experimental operation and data analysis were completed by two professionals. Our laboratory was evaluated by the National Reference Laboratory of China, and the positive standard strain *M. tuberculosis* H37Rv (ATCC27294) and the negative standard strain *Escherichia coli* (ATCC25922) were provided by the Chinese Center for Disease Control and Prevention.

We prepared nucleic acid templates of standard strains *E. coli* (ATCC25922) and H37Rv (ATCC27294) as double-well negative and positive controls. We used these with the negative and positive controls of commercial PCR amplification kit to participate in the whole experimental process, and monitor the experimental process, reagent quality, and the experimental environment and quality. The negative and positive standards included in the kit monitored the experimental process and its effectiveness. The nucleic acid templates of the standard strains were used to monitor the experimental process while monitoring the quality of the negative and positive standards of the kit (which acted as a third-party quality control).

### Inclusion and exclusion criteria

Sputum samples were collected from patients with confirmed, suspected and unexcluded TB, according to the diagnostic criteria for TB in the Health Industry Standard of the People’s Republic of China (WS288-2017). We included: smear-positive patients; smear-negative patients but with positive diagnosis by other means, such as imaging and Xpert MTB/RIF; patients who were diagnosed after effective anti-TB treatment; patients without etiological evidence but positive TB-related detection indicators such as TB antibodies, tuberculin test or tuberculosis-specific T cells; and patients without any relevant indicators of TB examination, but in whom TB could not be excluded. The demographic and relevant clinical information was recorded at the same time, and the quality of the specimens was judged according to the sputum characteristics and acid-fast smears. Patients in whom tuberculosis could be excluded after diagnosis. Meet inclusion criteria but repeat tester (exclude repeat subject based on name, gender, date of birth, regional distribution etc.)

### Data analysis

We stratified patients positive for MTBC and MDR-TB by region to detect spatial patterns of MTBC dissemination and resistance. Pearson’s χ^2^ test was used to detect the prevalence of MTBC and the distribution of MDR-TB between the main urban and county and township areas, so as to identify areas with high drug resistance. Study variables potentially associated with MDR-TB in the main urban and county township areas were included in a multivariate logistic regression model to identify associations with MDR-TB in the context of other covariates. The spatial distribution maps of different types of MDR-TB in the main urban area and the county and township areas under its jurisdiction were drawn to refine the differences in the prevalence patterns of MDR-TB in different regions. Odds ratios and 95% confidence intervals (95% CIs) were used as relevant measures, and *p* < 0.05 was considered statistically significant. Data analyses were conducted with STATA/SE version 15.1 (Stata Statistical Software, College Station, TX, USA).

## Results

From January 2020 to December 2022, a total of 18,504 patients with confirmed, suspected or unexcluded TB who visited designated TB hospitals participated in the study, including 3017 (16.3%) in the main urban area and 15,487 (82.7%) in the county and township areas. The proportion of cases that were newly diagnosed, male, aged < 61 years, and smear-negative in the main urban and county and township areas were 96.3% (2906) and 97.0% (15,108), 69.9% (2108) and 67.2% (10,406), 66.9% (2018) and 59.0% (9134), and 73.0% (2201) and 90.8% (14,059), respectively. In patients aged < 61 years, cases were concentrated between 41 and 60 years in both the main urban and county and township areas (Table 1).

**Table 1:** Demographic characteristics of study participants

	Region		
	Total *n* (%)	Main urban *n* (%)	County and townships *n* (%)
Total	18,504 (100)	3017 (16.3)	15,487 (82.7)
Treated			
new cases	17,924 (96.9)	2906 (96.3)	15,018 (97.0)
previously treated cases	580 (3.1)	111 (3.7)	469 (3.0)
Sex			
male	12,514 (67.6)	2108 (69.9)	10,406 (67.2)
female	5990 (32.4)	909 (30.1)	5081 (32.8)
Age Category			
> 60	6953 (37.6)	859 (28.5)	6094 (39.3)
< 61	11,154 (60.3)	2018 (66.9)	9134 (59.0)
0–14	274 (1.5)	22 (0.7)	252 (1.6)
15–24	1720 (9.3)	347 (11.5)	1373 (8.9)
25–40	2886 (15.6)	680 (22.5)	2206 (14.2)
41–60	6274 (33.9)	969 (32.1)	5305 (34.3)
unknown	397 (2.1)	140 (4.6)	257 (1.7)
Sputum smear			
negative	16,260 (87.9)	2201 (73.0)	14,059 (90.8)
positive	2244 (12.1)	816 (27.0)	1428 (9.2)
Test year			
2020	5942 (32.1)	1023 (33.9)	4919 (31.8)
2021	7123 (38.5)	1129 (37.4)	5994 (38.7)
2022	5439 (29.4)	865 (28.7)	4574 (29.5)

The positive rate of MTBC detection was 14.5% (2675), which was significantly higher in the main urban area than in the county and township areas (32.8% vs. 10.9%, *p* < 0.001). There was a higher rate of MTBC carriage in re-treated, male and smear-positive patients in the main urban and county and township areas. The ratio of MTBC positive rate was 2.6 and 3.7 in the retreatment groups/newly diagnosed, 1.1 and 1.3 in the male/female groups, and 8.3 and 17.0 in the smear-positive/smear-negative groups, respectively. The positive rate of MTBC was higher in patients aged > 60 years than < 61 years in the main urban area (38.5% vs. 31.8%, *p* < 0.001), but the opposite was found in county and township areas (9.9% vs. 11.8%, *p* < 0.001). In patients aged < 60 years in the main urban area, the positive rate of MTBC was mainly concentrated in those age 41–60 years, compared with 25–40 years in county and township areas (Table 2).

**Table 2:** Differences in MTBC prevalence between the main urban areas and the county and township areas

	Region			
	Total *n* (%)	Main urban *n* (%)	County and townships *n* (%)	*p* value
Total	2675 (14.5)	990 (32.8)	1685 (10.9)	< 0.001
Treated				
positive result of new cases	2413 (13.5)	902 (31.0)	1511 (10.1)	< 0.001
positive result of retreated cases	262 (45.2)	88 (79.3)	174 (37.1)	< 0.001
Sex				
positive result of male	1948 (15.6)	718 (34.1)	1230 (11.8)	< 0.001
positive result of female	727 (12.1)	272 (29.9)	455 (9.0)	< 0.001
Age Category				
> 60	936 (13.5)	331 (38.5)	605 (9.9)	< 0.001
< 61	1719 (15.4)	642 (31.8)	1077 (11.8)	< 0.001
0–14	16 (5.8)	5 (22.7)	11 (4.4)	< 0.001
15–24	275 (16.0)	97 (28.0)	178 (13.0)	< 0.001
25–40	527 (18.3)	213 (31.3)	314 (14.2)	< 0.001
41–60	901 (14.4)	327 (33.7)	574 (10.8)	< 0.001
unknown	20 (5.0)	17 (12.1)	3 (1.2)	< 0.001
Sputum smear				
positive	1818 (81.0)	748 (91.7)	1070 (74.9)	< 0.001
negative	857 (5.3)	242 (11.0)	615 (4.4)	< 0.001
Test year				
2020	910 (15.3)	338 (33.0)	572 (9.6)	< 0.001
2021	1042 (14.6)	391 (34.6)	651 (9.1)	< 0.001
2022	723 (13.3)	261 (30.2)	462 (8.5)	< 0.001

From 2020 to 2022, the overall detection rate of MDR was 10.1%, which was higher in the main urban area than in the county and township areas (13.9% vs. 7.8%, *p* < 0.001). The detection of MDR in newly diagnosed, male and smear-positive patients and those aged < 61 years was significantly different between the main urban and county and township areas. The differences in patients aged < 61 years were mainly in the 25–40 and 41–60 years age groups, whereas there were no significant differences in the re-treated and female patients, those aged > 60 and 15–24 years, and smear-negative patients (Table 3).

**Fig. 2:**
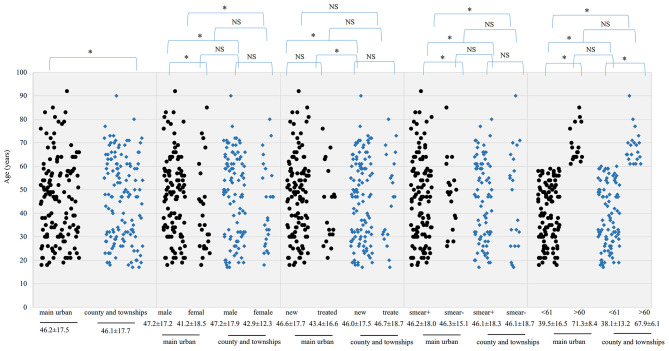
Scatter plot of age distribution of MDR among different populations between the main urban and the subordinate county and township areas. The dots indicate the age of patients of MDR and the median age is presented below the variables. ● and ♦ denote MDR group age distribution in main urban area and township area, respectively; * indicates statistical significance, NS indicates no statistically significant. *p*-value stander was 0.05

The average age of MDR-TB carriers was 46.2 ± 17.5 years in the main urban area and 46.1 ± 17.7 years in the county and township areas; 47.2 ± 17.2 and 41.2 ± 18.5 years for males and females in the main urban area; and 47.2 ± 17.9 and 42.9 ± 12.3 years in the county and township areas. The detection rate of MDR-TB was higher in males than females in the main urban areas (15.7% vs. 9.2%, *p* < 0.001), while there was no significant sex difference in county and township areas (8.5% vs. 5.7%, *p* = 0.055). The detection rate of MDR-TB in the retreatment group was significantly higher than that in the newly diagnosed group (14.5% vs. 9.6%, *p* = 0.012). However, there was no significant difference in the detection rate of MDR-TB between the retreatment and newly diagnosed groups in the main urban and county and township areas (20.5% vs. 13.3%, *p* = 0.065 and 11.5% vs. 7.3%, *p* = 0.053). The age range with the highest MDR-TB detection rate was 25–40 years in both the main urban and county and township areas. Patients aged < 61 years had a higher detection rate of MDR-TB than those aged > 60 years (16.6% vs. 8.5%, *p* < 0.001 and 8.9% vs. 5.8%, *p* = 0.022). The detection rate of MDR-TB was higher in smear-positive than in smear-negative patients in the main urban area, but there was no significant difference between these groups in the county and township areas (Table 3; Fig. 2).

**Table 3:** The difference of MDR detection between the main urban area and the county and township areas

	Region			
	Total	Main urban *n* (%)	County and townships *n* (%)	*p* value
Total	269 (10.1)	138 (13.9)	131 (7.8)	< 0.001
Sex				
MDR-TB in males	218 (11.2)	113 (15.7)	105 (8.5)	< 0.001
MDR-TB in females	51 (7.0)	25 (9.2)	26 (5.7)	0.076
treated				
MDR-TB in new cases	231 (9.6)	120 (13.3)	111 (7.3)	< 0.001
MDR of retreated cases (%)	38 (14.5)	18 (20.5)	20 (11.5)	0.052
Age Category				
> 60	63 (6.7)	28 (8.5)	35 (5.8)	0.119
< 61	201 (11.7)	105 (16.6)	96 (8.9)	< 0.001
0–14	0 (0.0)	0 (0.0)	0 (0.0)	/
15–24	32 (11.6)	15 (15.5)	17 (9.6)	0.144
25–40	80 (15.2)	42 (19.7)	38 (12.1)	0.017
41–60	89 (9.9)	48 (14.7)	41 (7.1)	< 0.001
unknown	5 (25.0)	5 (29.4)	0 (0.0)	/
sputum smear				
positive (%)	196 (22.9)	116 (47.9)	80 (13.0)	< 0.001
negative (%)	73 (4.0)	22 (2.9)	51 (4.8)	0.706
test year				
2020	96 (10.5)	46 (13.6)	50 (8.7)	0.021
2021	119 (11.4)	62 (15.9)	57 (8.8)	< 0.001
2022	54 (7.5)	30 (11.5)	24 (5.2)	0.002

In adjusted multivariate logistic regression models, treatment history, male sex and age < 61 years were all significantly and positively associated with an increased risk of MDR-TB. The detection of MDR-TB in the main urban and county and township areas was 1.80 (95% CI 1.13–2.89) and 1.58 (95% CI 1.01–2.48) times more likely in males than in females. In patients aged < 61 years, detection of MDR-TB was 2.10 (95% CI 1.35–3.28) and 1.64 (95% CI 1.09–2.45) times more likely than in those aged > 60 years. Re-treated patients were 1.65 (95% CI 1.14–2.40) times more likely than newly diagnosed patients to be diagnosed with MDR-TB. Detection of MDR-TB in re-treated patients in the main urban and county and township areas was 1.68 (95% CI 0.95–2.98) and 1.64 (95% CI 0.99–2.73) times more likely than in newly diagnosed patients, although the difference was not significant (Table 4).

**Table 4:** Logistic regression analysis of risk factors for MDR epidemic

	Total			Main urban			County and townships		
	*n* (%)	aOR (C I95%)	*p* value	*n* (%)	aOR (CI 95%)	*p* value	*n* (%)	aOR (CI 95%)	*p* value
treated									
new cases (%)	231 (9.6)	reference		120 (13.3)	reference		111 (7.3)	reference	
previously treated cases (%)	38 (14.5)	1.65 (1.14,2.40)	0.009	18 (20.5)	1.68 (0.95, 2.98)	0.072	20 (11.5)	1.64 (0.99,2.73)	0.056
Sex									
male (%)	218 (11.2)	1.67 (1.21,2.30)	0.002	113 (15.7)	1.80 (1.13, 2.89)	0.014	105 (8.5)	1.58 (1.01,2.48)	0.044
female (%)	51 (7.0)	reference		25 (9.2)	reference		26 (5.7)	reference	
Age Category									
*> 60*	63 (6.7)	reference		28 (8.5)	reference		35 (5.8)	reference	
*< 61*	201 (11.7)	1.87 (1.39,2.52)	< 0.001	105 (16.6)	2.10 (1.35, 3.28)	0.001	96 (8.9)	1.64 (1.09,2.45)	0.017
sputum smear									
negative (%)	73 (4.0)	reference		116 (47.9)	reference		80 (13.0)	reference	
positive (%)	196 (22.9)	1.29 (0.97,1.72)	0.084	22 (2.9)	1.81 (1.10, 2.97)	0.020	51 (4.8)	0.91 (0.63,1.31)	0.606
test year									
2020	96(10.5)	reference		46 (13.6)	reference		50 (8.7)	reference	
2021	119 (11.4)	1.13 (0.85,1.51)	0.391	62 (15.9)	1.25 (0.82,1.90)	0.301	57 (8.8)	1.07 (0.71,1.60)	0.747
2022	54 (7.5)	0.65 (0.45,0.93)	0.019	30 (11.5)	0.74 (0.44,1.25)	0.254	24 (5.2)	0.61(0.37,1.01)	0.053

*aOR* adjusted odds ratio, *CI* confidence interval

The “reference” labeled in “Table 4” indicates that this group is used as the reference category, and whether the other group is a risk factor for the occurrence of drug resistance compared with this group, which is used to evaluate the risk of drug resistance in the other group relative to this reference group

**Fig. 3:**
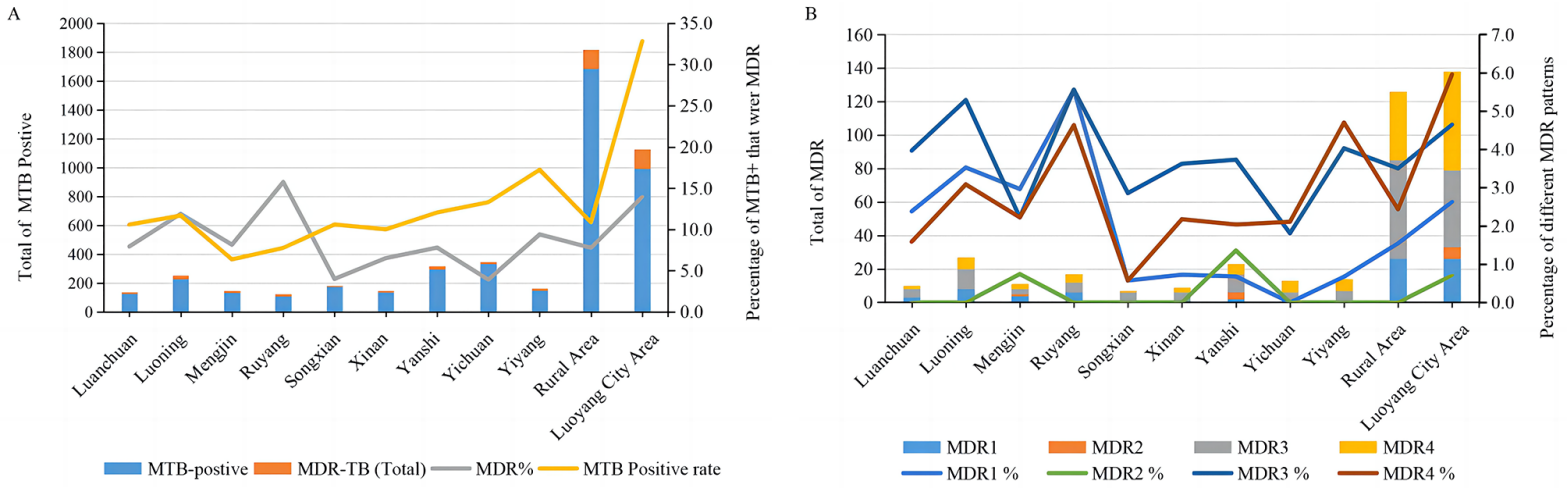
Spatial distribution of different resistance patterns of MDR. **A** Spatial distribution of MTBC positive and MDR-TB in Luoyang area; **B** Spatial distribution of different resistance patterns in MDR-TB; MDR1 INH þ RIF, MDR2 INH þ RIF þ EMB, MDR3 INH þ RIF þ SM, MDR4 INH þ RIF þ EMB þ SM. INH isoniazid; RIF rifampicin; EMB Ethambutol; SM Streptomycin;

Yiyang had the highest MTBC positive rate at 17.2% (149), followed by Yichuan and Yanshi at 13.3% (332) and 12.1% (295), respectively, and Mengjin had the lowest rate at 6.4% (135). Ruyang was the region with the highest MDR-TB detection rate at 15.7% (17), followed by Luoning and Yiyang at 11.9% (27) and 9.4% (14), respectively. Yichuan and Songxian had the lowest rate at 3.9% (13) and 4.0% (7) (Fig. 3A). MDR3 (INH þ RIF þ SM) and MDR4 (NH þ RIF þ EMB þ SM) were the main resistance patterns in this region, followed by MDR1 (INH þ RIF) and MDR2 (INH þ RIF þ EMB), with positive rates of 3.9% (105), 3.7% (100), 1.9% (52) and 0.3% (7), respectively. The detection patterns of MDR-TB differed between the main urban and county and township areas. MDR4 (NH þ RIF þ EMB þ SM) was predominant in the main urban area, followed by MDR3 (INH þ RIF þ SM), MDR1 (INH þ RIF) and MDR2 (INH þ RIF þ EMB) with positive rates of 6.0% (59), 4.6% (46), 2.6% (26) and 0.7% (7), respectively. In the county and township areas, MDR3 (INH þ RIF þ SM) was dominant, followed by MDR4 (NH þ RIF þ EMB þ SM) and MDR1 (INH þ RIF), with positive rates of 3.5% (59), 2.4% (41) and 1.5% (26), respectively, and no MDR2 (INH þ RIF þ EMB). The drug resistance pattern differed among county and township areas. For example, the detection rate of MDR3 (INH þ RIF þ SM) in Luanchuan was the highest, followed by MDR1 (INH þ RIF), with positive rates of 4.0% and 2.4%, respectively, and no MDR2 (INH þ RIF þ EMB). Mengjin was dominated by MDR1 (INH þ RIF) with a positive rate of 3.0%. Yiyang was dominated by MDR4 (NH þ RIF þ EMB þ SM) and MDR3 (INH þ RIF þ SM), with positive rates of 4.7% and 4.0%, respectively, while Yichuan only had MDR4 (NH þ RIF þ EMB þ SM) and MDR3 (INH þ RIF þ SM), with positive rates of 2.1% and 1.8%, respectively (Fig. 3B).

## Discussion

The continued decline in TB cases is testament to the remarkable success of the global anti-TB program, but the gap between new and reported TB cases was estimated to be about 3 million in 2019, whis is a warning that the continued decline in reported TB may be partly due to under-reporting and under-diagnosis [15]. The COVID-19 pandemic was bound to exacerbate such shortcomings and directly affected the outcome of TB diagnosis and treatment [16], which is not conducive to accurate assessment of the current epidemic situation of TB and its prevention and control. Therefore, we investigated the epidemiology of TB in the local area during the COVID-19 pandemic, to gain an overall view of the prevalence of TB and multidrug resistance, and provide support for the overall planning of local anti-TB measures.

During the study period, the local rates for MTBC and MDR-TB showed a downward trend, which is beneficial for local TB control strategies and improvement of treatment capacity; however, the impact of COVID-19 prevention and control measures cannot be ignored [17]. From 2020 to 2022, the number of confirmed, suspected and unexcluded TB cases in designated hospitals was 121/100,000 in the main urban area and 338/100,000 in the county and township areas, and the ratio of the number of MTBC-positive cases in the county and township areas to the main urban area was 1.7. In China, about 71% of TB patients live in rural areas [18], and the large population base, shortage of public health resources, and poor medical facilities are disadvantages to the implementation of TB prevention and control [19]. In recent years, China’s economic reforms and urbanization have lifted many non-urban residents out of poverty and some have become a new type of urban dweller, but their benefits in urban health insurance are limited due to the household registration system (hukou system) [20]. In addition, due to the limitations of education and other factors, their work is mainly manual labor, and these socioeconomic barriers can also lead to a higher risk of TB compared with urban residents [21].

The detection rate of MTBC in the main urban area was significantly higher than in the county and township areas (32.8% vs. 10.9%) in patients attending designated TB hospitals. Due to the gap in medical level, the control of screening criteria for TB patients in county and township areas is not strict, and the quality of sputum samples is low. This was demonstrated by the smear-positive rate in different regions, which was 27.0% in the main urban area and only 9.2% in the county and township areas. The latter areas even lack the necessary infrastructure for TB screening, which may lead to some cases not being reported due to insufficient diagnosis and treatment. In addition, the main urban area has a robust TB reporting system, more abundant diagnostic resources, and more convenient medical institutions, making TB diagnosis and reporting more rapid and timely.

The spread of MTBC is a complex process, and factors such as household registration policy, age, sex and nutritional status can lead to differences in characteristics of TB patients [22]. Local TB epidemics and people with a high burden of MDR-TB are concentrated in male and re-treated patients and those younger than 61 years. Therefore, MTBC screening in TB-designated hospitals should be considered in these groups in order to control the spread of TB more effectively.

The overall positive rate of MTBC was 14.5% and 13.5% in the newly diagnosed group, which is higher than the 55/100,000 people announced by the WHO in 2021 [1], indicating that the prevention and control of TB has a long way to go. The incidence of TB is significantly higher in males than in females, and in 2018, WHO reported that the global TB burden was 56% in adult males and 32% in adult females [23]. The male to female ratio of TB cases in our study was 2.7, which may be related to a wider range of activities, more physical exertion, and poor lifestyle habits among males [24]. The prevalence of MTBC in designated TB hospitals in the main urban area was mainly concentrated in the group aged > 60. In this age group, the urbanization process is accelerated, the aging population is serious, and more susceptible to TB because of factors such as low immunity and malnutrition [25, 26]. The opposite is true for county and township areas, where the higher prevalence of MTBC was in individuals aged < 61 years, which may be because they have good mobility. They are the main economic providers for their families; they often shuttle around cities to seek employment opportunities, and their main working space is confined and crowded, and the relatively strong awareness of active visits by county and township young people in the new era may be another reason. Therefore, while focusing on TB in older people, we also need to consider the increasing incidence in younger people, and whether MTBC needs to be included in screening for respiratory pathogens in high-risk groups.

The positive rate of MMCA detection was 81.0% in the smear-positive and 5.3% in the smear-negative patients. Molecular diagnosis is considered to be more time-efficient and has higher sensitivity and specificity than traditional methodology [27]. The recent widespread application of molecular detection techniques has sometimes resulted in their diagnostic role being over-emphasized, while ignoring their limitations, such as being susceptible to antimicrobial drugs and prone to false-positive results. Also, the commercial kit used in this study is not able to detect nontuberculous mycobacteria. This means that attention should be paid to combination of traditional and new methods for diagnosis and treatment of TB. In the smear-positive patients, the positive rate of MTBC was higher in the main urban area than in the county and township areas (91.7% vs. 74.9%), but in the smear-negative patients, it was lower in the county and township areas than in the main urban area (4.4% vs. 11.0%). This indicates that the medical institutions in the county and township areas had excellent performance for exclusion of TB, but there was a difference in the confirmed diagnosis of TB compared with the main urban area, so it is necessary to pay attention to the false positives of tuberculosis diagnosis for confirmed cases reported by smear-positive as a detection method in county and township areas.

The detection rate of MDR-TB in the main urban area was significantly higher than that in the county and township areas. Due to the particularity of medical policies (The registered permanent residence system in China has little impact on the treatment of confirmed cases), confirmed cases of TB in different residences, especially some complex, refractory and unsuccessful treatment cases were concentrated in the main urban area with relatively abundant medical resources, which is a major reason for the high MDR detection rate in the main urban areas [28]. In addition, high mental stress, poor medication compliance, irregular treatment, drug abuse, and direct spread of MDR strains [29–31] are all reasons for the high drug resistance of MTBC in the main urban area.

In 2019, there were 361,920 MDR-TB cases worldwide, and these may have arisen through secondary acquisition of resistance [32]. In our study, the MDR-TB rate in the local re-treated population was 45.2%, while it was as high as 79.3% in the main urban area. A history of TB treatment and irregular treatment resulted in higher detection of MDR-TB in this group compared with the newly diagnosed group [33].

The prevalence and drug resistance of TB vary regionally, with female and young groups at high risk of MDR-TB in some areas, while older and male groups are strongly associated with MDR-TB in other regions [15]. The detection of TB in the main urban area was concentrated in the 41–60 years age group, compared with 25–40 years in the county and township areas. Overall, individuals with a history of TB treatment, male sex, and age < 61 years were high-burden groups for MDR-TB in our study region. However, none of the differences in the detection of MDR-TB were significantly affected by the presence or absence of treatment history among TB cases in the main urban and county and township areas, and more data are needed to elucidate the causes.

Our study had several limitations. First, study participation was voluntary, which may have led to missed detection of some hidden cases of TB or the failure of some patients to be fully monitored due to economic and other reasons. There were insufficient data for some particular groups of TB patients, such as older patients in remote areas, patients with poor economic conditions, and patients who have less understanding of TB and reluctance to visit the doctor. These factors could have led to bias in the study of TB in our area. Second, some sputum specimens were of low quality, which could have affected the final MTBC detection rate, and led to some false-negative results and a lower TB infection rate than is actually present.

Despite the shortcomings, we covered a wide area of Luoyang City, and we believe that our results do reflect the epidemiology of MTBC and its drug resistance characteristics in local urban and county and township areas. Our findings may be applicable to TB epidemics across the whole of China during the particular period of the COVID-19 pandemic but not in areas with high TB incidence, large-scale outbreaks, and poorly controlled TB.

## Conclusions

The epidemiology of MDR-TB varies according to factors such as regional distribution, education level, gaps in medical conditions population migration, economic level and population mobility. The epidemic manifestations of MDR-TB in the main urban and county and township areas were different, and different jurisdictions in the county and township areas had different drug-resistance patterns. This suggests that in our anti-tuberculosis career, we need to clarify the differences in MTBC infection in different regions according to the actual situation. There is a need to adapt TB control to local conditions, and choose measures according to the differences in MDR-TB patient characteristics and regional distribution, and the unique drug-resistance patterns in the region to achieve the goal of the Ending TB Strategy in China.

## Data Availability

The original contributions presented in the study are included in the article/supplementary material. Further inquiries can be directed to the corresponding authors.

## Abbreviations

MTBC: *Mycobacterium tuberculosis* complex
TB: Tuberculosis
MDR: Multidrug resistance
MDR-TB: Multidrug resistance tuberculosis
COVID-19: Coronavirus disease 2019
MMCA: Multicolor Melting Curve Analysis
INH: Isoniazid
RIF: Rifampicin
EMB: Ethambutol
SM: Streptomycin
WHO: World Health Organization
